# Integrated Design and Simulation of Tunable, Multi-State Structures Fabricated Monolithically with Multi-Material 3D Printing

**DOI:** 10.1038/srep45671

**Published:** 2017-03-31

**Authors:** Tian Chen, Jochen Mueller, Kristina Shea

**Affiliations:** 1 Engineering Design and Computing Laboratory, D-MAVT, ETH Zurich, Switzerland

## Abstract

Multi-material 3D printing has created new opportunities for fabricating deployable structures. We design reversible, deployable structures that are fabricated flat, have defined load bearing capacity, and multiple, predictable activated geometries. These structures are designed with a hierarchical framework where the proposed bistable actuator serves as the base building block. The actuator is designed to maximise its stroke length, with the expansion ratio approaching one when serially connected. The activation force of the actuator is parameterised through its joint material and joint length. Simulation and experimental results show that the bistability triggering force can be tuned between 0.5 and 5.0 N. Incorporating this bistable actuator, the first group of hierarchical designs demonstrate the deployment of space frame structures with a tetrahedron module consisting of three active edges, each containing four serially connected actuators. The second group shows the design of flat structures that assume either positive or negative Gaussian curvature once activated. By flipping the initial configuration of the unit actuators, structures such as a dome and an enclosure are demonstrated. A modified Dynamic Relaxation method is used to simulate all possible geometries of the hierarchical structures. Measured geometries differ by less than 5% compared to simulation results.

An active or smart structure initiates state or function changes to respond to external stimuli. Such behaviours are observed both in nature and in human-made artifacts. Mechanically active structures have seen applications that largely fall into two categories: deployment and energy absorption. Deployment involves transforming a device between desired states; common applications are in robotics^1^ and morphing structures^2^. Energy absorption and propagation is a newly investigated field where an active structure deforms predictably under impact and either absorbs or transmits kinetic energy^3^,^4^.

Bistability is one increasingly popular route towards achieving form or functional changes within a structure. One can group such structures by their applications in either energy- or motion-related fields^5^. Impact absorption^3^,^6^,^7^ is one energy-related field that has seen increased attention. Examples of motion-related applications may include robotic manipulators, active building facades, large scale space structures^2^ and trapping mechanisms^8^.

From reviewed literature, complex activation is achieved through the tiling of multiple bistable units. With morphing, Schioler and Pellegrino^2^ convert a static truss to an active one by replacing some of the members using bistable actuators. Utilising 3D printing, Restrepo *et al*.^3^ and Shan *et al*.^7^ connected multiple bistable mechanisms to form 2D energy absorbing blocks. Han *et al*.^9^ proposed a quad-stable mechanism and commented on the parameters influencing the structure, in particular the rigidity of the frame constraining the buckling elements. In Oh and Kota^10^, a rotational quad-stable mechanism was proposed. It shows a hierarchical structure where the outer bistable mechanism uses the inner one as a pin. Oh and Kota also stated that by designing the mechanism in a single layer, it can be manufactured in one piece cost effectively.

An attractive feature of bistability is that energy input is only needed to transform between states, but not to maintain them. Using a bistable mechanism is novel compared to others who use swelling or prestressing to achieve large magnitude activation. Mao *et al*.^11^ utilise the shape-memory effect of thermal plastics to “lock-in” pre-stress, and achieve shape change through temperature variation. Raviv *et al*.^12^ achieve large state changes through swelling of strategically printed hydrogels.

Both Mao *et al*.^11^ and Raviv *et al*.^12^ fabricate their designs using a multi-material inkjet 3D printer able to deposit materials with varying stiffness. These and additional works connecting 3D printing and activation were reviewed by Choi^13^ who noted significant reduction in transportation volume and weight when compared with conventional assemblies. However, shortcomings are that the designs do not demonstrate predictable reversibility, the activated state cannot be precisely controlled and, there is no defined load bearing capability.

In this work, we use the same 3D inkjet printing technology as Mao *et al*.^11^ and Raviv *et al*.^12^ to fabricate proof-of-concept deployable structures that overcome the aforementioned challenges. Utilising hierarchical principles^14^, we start by designing a unit actuator based on the Von Mises Truss (VMT)^15^. Then, we create deployable structures using multiple instances of the proposed actuator. The resulting designs possess multiple equilibrium states, are fabricated flat and activated to 3D structures.

These characteristics are important in many engineering applications. The deployment of space lattice structures^2^ and masts^16^ can greatly benefit from the ability to be transported flat or in a coil to drastically reduce volume. Active reconfiguration and reversibility may be adopted in shape changing structures such as building facades^17^, PV arrays^18^ and implants for better fit. As concrete examples, we demonstrate the deployment of a dome that may be used as a temporary structure and an enclosure unit that may be used in drug delivery. This work extends the state of the art in 3D printed, reconfigurable and deployable structures that save printing material and time as well as provide the foundation for novel self-assembly and activation.

The paper begins by describing the unit actuator with its simulation and experimentation results. Next, we use this unit actuator to design a deployable, tetrahedral space frame based on hierarchical principles. Last, more complex structures are presented to demonstrate different deployment configurations. A modified Dynamic Relaxation simulation method is employed to find their activated states. Material characterisation, simulation and experimental procedure are described in the SI.

## Methods

### Design of the Bistable Unit Actuator

**Figure 1:**
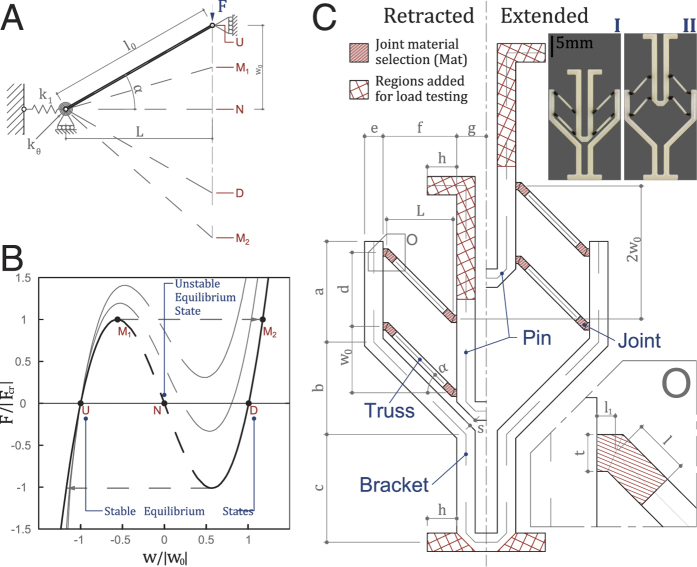
(**A**) VMT consisting of a truss member with geometric variables and critical points labeled, (**B**) Possible force displacement curves of a VMT, (**C**) Drawing of the proposed bistable unit actuator. The line-shaded region (zoomed in O) indicates the location of the joint and a change in material stiffness. The length of the joint *l* = 1.0, 0.75, 0.50 mm is defined parametrically. Note that changing *l* does not change the overall geometry, i.e. if the joint lengthens, the truss member itself shortens. The cross-hatched regions indicate the additional structure added to facilitate load testing. These regions are removed in the designs presented in latter sections. A benchmark actuator (*l* = 0.75, joint material F9860) is fabricated (I) and activated (II). Parameter values are listed in SI. Table 2.

The goals of the unit actuator design are: (1) it must use bistability, (2) the stroke length should be maximised and (3) the critical force should be made adjustable and predictable. In relation to the overarching goal of printing deployable structures, the unit actuator itself must be printable as a flat part and allow for serial and parallel connection. A bistable structure exploiting the limit point buckling behaviour is proposed in this work. The Von Mises Truss (VMT) was first introduced in 1923^15^ as a structure that features two pin jointed truss members with a vertical load at the apex (Fig. 1A). A geometrically non-linear solution offers force displacement diagrams as shown in Fig. 1B^19^.

In Fig. 1A,B, points *U* and *D* are the equilibrium states when the load *F* is zero. *M*_1_ is the critical point at which an infinitesimal increase in load triggers instability. With this increase in load, the structure snaps, after which the VMT mirrors itself along the horizontal axis. With a further increase in load, the members experience tension and eventually rupture. If the load is displacement controlled, past point *M*_1_, the reaction force decreases and eventually reverses orientation past point *N* and reaches a minimum between point *N* and *D*. Note that the symmetric behaviour above is achieved only if the stiffness of the rotational spring is zero, *k*_*θ*_ = 0. The thinner curves in Fig. 1B show possible asymmetric behaviours^20^. Schioler *et al*.^2^ and Shan *et al*.^7^ have physically realised such a bistable actuator at different scales and using different fabrication processes.

Schioler stated that compared to an Euler buckling based bistable mechanism (e.g. Restrepo *et al*.^3^ and Chen *et al*.^21^), the stroke length provided by VMTs is much smaller due to the large strain induced in the joints. To overcome this limitation and to accomplish the aforementioned goals, we propose the bistable actuator shown in Fig. 1C. The design consists of a bracket, four trusses and a pin. Two vertically stacked VMTs ensure that all DOFs except in the vertical direction are restricted^2^,^22^. The bracket, the pin and the centre portion of the trusses are fabricated with a rigid plastic, and are much stiffer than the joints, which are fabricated with a compliant elastomer. The inclination angle of the truss members is set to *α* = tan(*w*_0_/L) = 45°, giving a joint rotation of 90°. This value is below the stability limit^19^.

**Figure 2:**
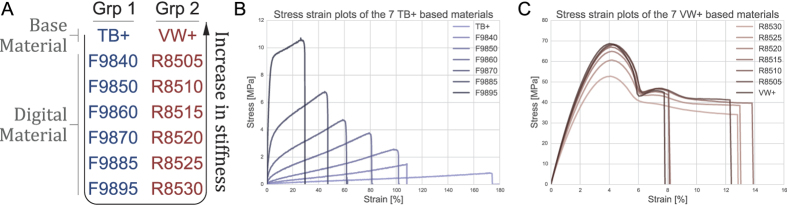
Available materials of the multi-material Printer. TB+ and VW+ are the base materials representing the most compliant and stiffest ends of the spectrum respectively. The 12 digital materials created at the time of printing with different mixture of TB+ and VW+ are shown. Direction of stiffness increase is indicated.

To fabricate this multi-material design, we utilize an inkjet 3D printer. By jetting a mixture of an elastomer-like and a rigid-plastic liquid photopolymer at different ratios, the printer is able to provide 14 materials of varying mechanical properties. At the extreme ends are two base materials named TangoBlackPlus (TB+), and VeroWhitePlus (VW+). Designation of the intermediate digital materials are shown in Fig. 2A. The stress strain behavior of the DMs are obtained as a pre-study and are plotted in Fig. 2B,C. The experimental work behind these plots are detailed in the SI.

The DMs from material behavior shown in Fig. 2B is used to achieve a joint rotation of 90°. With this value, the stroke to length ratio of serially connected actuators approaches 100%. This is significantly larger than bistable designs using the Euler buckle shape, e.g. Restrepo *et al*.^3^ at 16%, or ones based on VMTs, e.g. Schioler and Pellegrino^2^ at 29%, Barbarino *et al*.^23^ at 24% and Haghpanah *et al*.^24^ at 20%.

With the stroke length maximised, we parametrise the joint material and the joint length *l* to study their effect on the overall behaviour and the activation force. With such an actuator design, it is seen that the necessary conditions for symmetric behaviour is not met (Fig. 1B). Further, the two stacked VMTs would experience different support stiffness. These complexities lead us to perform numerical and physical experiments on a series of 42 models. The parametric variations include seven joint materials (Fig. 2B and SI. Table 1), three length variations *l* = 1.0, 0.75, 0.50 mm, and two initial configurations, i.e. retracted and extended. Five sets of identical specimens are fabricated and tested to validate the numerical results.

### Design of Hierarchical Structures

Surveyed literature show that multi-stable designs are achieved through clever stacking of bistable units. Similar to springs, one may connect bistable units in series, in parallel or both^25^. Concepts from Han *et al*.^9^ and Oh *et al*.^10^ regarding frame rigidity, hierarchical design and flat printing are further developed in this work, where a unit actuator is used as the basic building block in the design of complex multi-stable hierarchical structures. The goals are to create load bearing structures that may be transported as flat sheets and have predictable deployed states.

Since the proposed unit actuator has two equilibrium states, a multi-stable structure can be achieved by integrating multiple unit actuators hierarchically. Providing that each actuator can be activated within the overall structure, the resulting geometry is guaranteed to be independent of the activation force. Therefore, one can start integrating the proposed actuator in structures geometrically first and then consider the activation aspect. Similarly, if a different stroke length is desired, one only needs to adjust the inclination angle *α* of the truss members. To enable a serial connection between actuators, the bracket is designed to be identical to the pin (Fig. 1C). In this way, the “wasted-length” between the bottom of the pin and of the bracket is eliminated. Parallel connection is enabled by connecting the bracket and the pin of two or more units.

**Figure 3:**
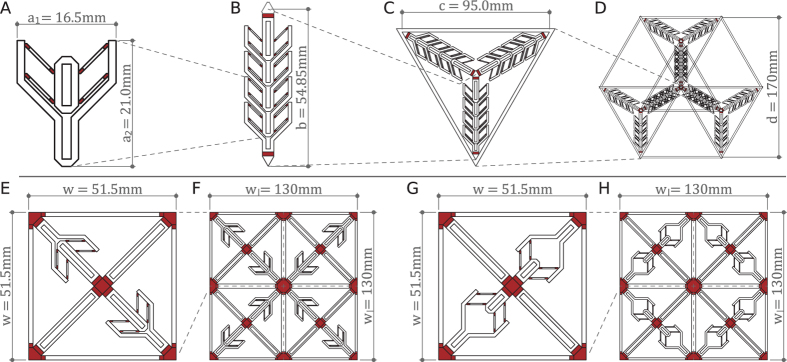
Designs of proof-of-concept hierarchical structures. (**A–D**) Show the design of a tetrahedral space frame outlining the hierarchy from a single unit actuator (A) to a tessellation of tetrahedral modules (D). (**E–H**) Show the grid-based hierarchical designs using the same unit actuator (A) to build the square unit modules (E,G) and the tiling of these modules (F,H).

The first example of hierarchical design stems from the construction of space frames and utilizes serial connectivity of the unit actuators. A typical space frame can be defined with the edges from a tetrahedral tessellation of a given volume. Using this set of edges, one guarantees that the space frame is a load bearing truss structure. The levels of the design hierarchy from a single unit actuator to a tessellation of the tetrahedral modules are shown in Fig. 3A–D. A regular tetrahedron with *c* as the length of each edge is used. The tetrahedral module consists of three edges of parallel actuators (Fig. 3C). When folded, these apex-connecting edges must retract into the base. Therefore, their length equals the radius of circumscribing circle of the base triangle *b* (Fig. 3B). We also demonstrate that this module can be tiled to form larger load bearing structures (Fig. 3D).

The second example demonstrates the versatility of hierarchical design to achieve complex state change. A unit module using the same unit actuator is printed in retracted or extended configurations. The unit modules consist of two actuated and two rigid cross members in a flexible frame assuming pinned connections (Fig. 3E,G). These modules are tiled to form grid surfaces that can assume Gaussian curvatures in both the positive and the negative ranges with simple initial orientation and configuration changes of the unit actuator. Such structures (i.e. 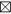) are not statically determinate. When activating the actuators, internal stress develops in the rigid members in a planar analysis. However, as the joints accommodate out-of-plane rotation, the unit modules assume an activated shape in the third dimension. These structures cannot be activated with a single activation load, the activated state must be obtained with simulation and there exists multiple, non-symmetrical activated states.

As demonstrated by these two examples, the number of equilibrium states grow exponentially with each added unit actuator, we propose a generalized kinematics based simulation to determine the activated states of each design. Such a form finding method drastically reduces the computation effort as compared to FEM. It assumes that each actuator can be activated independently. This assumption remains valid as long as the structure is determinant, i.e. there is no self-stress preventing activation. In literature, form finding of actuated structures has been done by explicitly deriving the geometrical relationships between the vertices^26^. As a different derivation is needed for each initial configuration, the practicality of this method is limited to simple structures. In this work, a modified Dynamic Relaxation (mDR) method is used to find the activated states^27^. The modifications include iteratively updating the Force Density Matrix based on the difference between current member length and the activated member length^8^. For 2D to 3D deployment as shown in the presented examples, an initial perturbation is introduced in the form of a small velocity at time zero. By iterating through all *S* = 2^*n*^ combinations of bistable states, one is able to obtain all unique activated states using the mDR. Additional states can be found in the case of 2D to 3D deployment by changing the initial velocity based perturbation. These states result from mirroring of the unit modules. A detailed description of the mDR used in this work is included in the SI.Section - Simulation using Modified Dynamic Relaxation.

## Results

### Unit Actuator Simulation and Experiments

**Figure 4:**
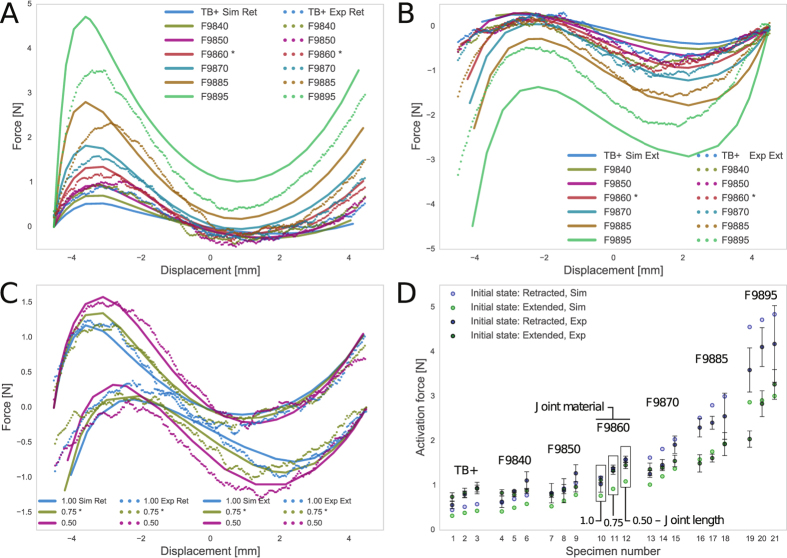
Simulation and experimental force-displacement graphs. (**A,B**) Includes specimens with fixed joint length but varying material. This shows that 1) an increase in joint stiffness increases the activation force, 2) simulation shows agreement with experimental data, and 3) the extended initial configuration attains lower activation force than the retracted initial configuration. (**C**) Includes specimens with fixed joint material stiffness but varying joint length and shows that a decrease in joint length increases the activation force. (**D**) Shows the critical force attained by each specimen (see SI. Table 3 to identify the specimens). *Denotes the benchmark configuration.

A subset of the 42 specimens are shown in Fig. 4 to assess the bistable actuator’s overall behaviour. This subset of 12 expands from the benchmark model and includes all specimens with *l* = 0.75 or is fabricated with F9860. Figure 4A–C show that bistable snap-through behaviour is observed with all specimens. Regarding the overall behaviour, the increase in material stiffness translates to increase in actuator stiffness. Simulations show excellent agreement with the experimental results with joint material F9860 at all joint lengths *l* = 1.0, 0.75, 0.50 mm (Fig. 4C), differing by 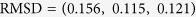 for the retracted configuration and 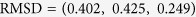 for the extended. Simulation overestimates the strength of higher stiffness joints. This is linked to the fact that experimental error also increases. As the stiffness of the joint material increases, structural behavior becomes more dependent on the performance of the interface between the joint and the surrounding parts, i.e. the joint itself is no longer the “weakest-link”. It has been shown that interface regions are most prone to failure in multi-material 3D printing due to the inherent fabrication method^28^. This causes the increase in both experimental error and deviation from simulation, as the joint interface is assumed to remain perfect under any deformation in finite element simulation.

Increase in activation force is observed with an increase in joint stiffness printed in both retracted and extended configurations. The activation force also increases with a decrease in joint length (Fig. 4D). Both observations agree with the expected behaviour. With the specimens printed in the retracted configuration, an activation force ranging from 0.50 N to 5.0 N can be achieved. With the specimens printed in the extended configuration, the activation force range is 0.25 N to 3.5 N.

The resulting force-displacement behaviour is not symmetrical about *w* = 0 or path independent (i.e. differences between the retracted and extended initial configuration are observed). This shows that both *k*_1_ and *k*_*θ*_ in Fig. 1B are finite. Further *k*_1_ of the upper VMT is less than that of the lower. This is expected as the bracket is more flexible further away from the base support. Similar behaviours are presented in literature^2^,^7^. One significant outcome of asymmetry is that the deployed equilibrium state does not reach full stroke length. The same observations can be made with the remaining 24 specimens.

### Hierarchical Structures

**Figure 5:**
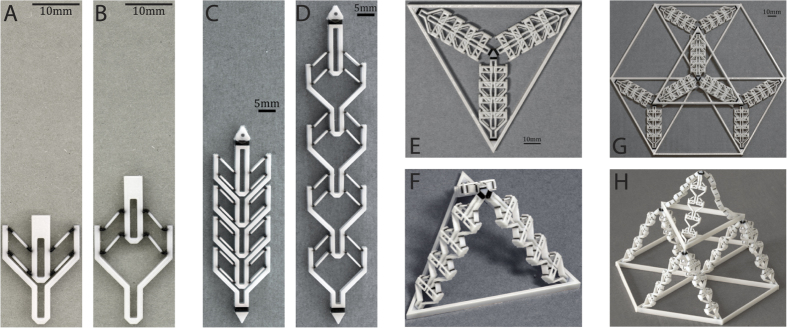
Design hierarchy from a single unit actuator (**E,F**) to a tessellation of tetrahedral modules (**G,H**). (**A,B**) Shows the unit actuator. (**C,D**) Shows four serially connected actuators. Note that the bracket has the same geometry as the pin. (**E,F**) Shows the tetrahedron module and the global joints used to connect the members. (**G,H**) Shows the tiling of multiple tetrahedron units to demonstrate the deployment of a space frame. The three corners of the top tetrahedron each connect with the apex of the three lower tetrahedra.

The tetrahedral specimens from the first example shown in Fig. 5 are fabricated flat and use minimal support material. As shown in Fig. 5C→D, the four actuators connected in series activate predictably with a tensile point load at the top. Post deployment, each actuated member lengthens from *b* = 54.85 mm to *c* = 95.00 mm. The measured lengths are 55 mm (Error: 0.27%) and 90.5 mm (Error: 4.8%) for *b* and *c* respectively. The error in *b* is negligible as the printer has a resolution of ±0.22 mm in the X-Y plane. With zero post-processing, one is able to activate both the tetrahedral module and the tessellation of modules with an upward force at the apex (Fig. 5E→F,G→H). The tetrahedral module is designed to attain an apex height of 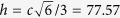
mm, where the measured height is 72.0 mm (E: 7.4%). The overall height of the tetrahedron tessellation is 155.0 mm and is measured as 142.5 mm (E: 8.4%).

**Figure 6:**
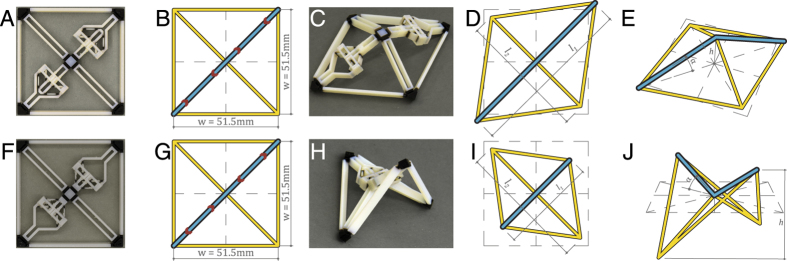
A unit module consisting of two actuators in either the retracted (**A**) or the extended (**F**) configuration (50 by 50). (**B,G**) Shows the initial simulation configuration. (**C,H**) Is the activated state of (**A,F**), resulting in a positively or negatively curved surface respectively. (**D,I,E,J**) Shows the simulation results in plan and isometric views, with geometrical parameters referred to in Table 1.

**Figure 7:**
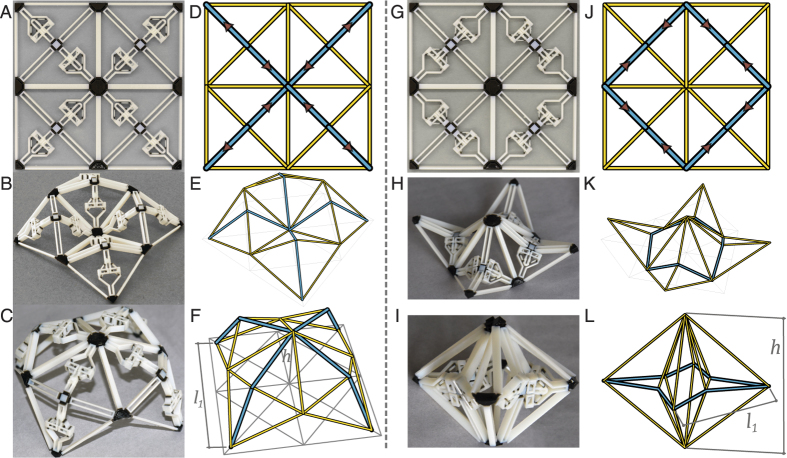
The grid structure with actuators in the retracted (**A**) or the extended (**G**) configuration. Two activated states are shown for each structure. The first resemble tiling of the activated unit modules (**B** and **H**), and the second show global behaviour that form a dome (**B**) or an enclosure (**H**). Simulation is shown in (**E,F,K,L**) where the yellow members are rigid trusses, and the blue members are actuated trusses.

In the grid-based hierarchical design example, first the square unit modules are fabricated and activated as shown in Fig. 6. Observe that by switching the initial configuration of the actuators, the activated geometry becomes either positively or negatively curved (Fig. 6C,H). Hierarchy is achieved by tiling these square unit modules in a 2 by 2 grid and restricting designs to those that are symmetric in both X and Y directions as shown in Fig. 7. Both unit modules and the grid structures are activated through sequential mechanical activation of the actuators. No self-stress is developed after each actuator activation, which in itself is an equilibrium state. Intermediate states are omitted and the structures are presented after all unit actuators are activated.

Tiling of the unit module shown in Fig. 6A results in a structure with two non-mirrored activated states (Fig. 7A→B,B→C). It is noted that one activated configuration (Fig. 7B) reassembles the tiling of the activated state of multiple square unit modules (Fig. 6C). However, in Fig. 7C, one observes that the unit module forms a part of a larger structure which in this case reassembles a dome. Tiling of the unit modules (Fig. 6F) results a structure shown in Fig. 7G→H,G→I). The first activated configuration can also be assembled using individually activated unit modules (Fig. 7H) and the geometry is similar to large span concrete hypar roofs. Global behaviour is observed in Fig. 7I. The four unit modules fold inward to form an enclosed volume.

## Discussion

With the above proof-of-concept structures, we demonstrate the principle of 3D printed, hierarchical deployable designs utilizing a bistable unit actuator. We show that the serial connection extends the total stroke length without increasing the activation force, whereas the parallel connection increases the activation force without changing the stroke length. As the activated state is in equilibrium, its geometry is easily predictable. This is of significant importance as precise deployment is mandatory in many applications. For the tetrahedron, since the initial configuration has no real thickness, we quantify deployability as an expansion ratio *e*_r_ between the activated height, *h*, and the side length, *c*. This ratio is predefined for a regular tetrahedron and is equal to 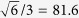
%. The error in activated geometry is due to the asymmetrical force displacement curve of the actuator, leading the deployed equilibrium to not attain full stroke length. This error accumulates as more actuators are stacked in series.

As shown in Table 1, mDR is capable of effectively obtaining the activated states. A detailed finite element simulation of the entire activation sequence would be computationally prohibitive, especially if multiple activated states are desired. Nevertheless, the discrepancy between the physical specimens and mDR is attributed to gravity and the finite dimensionality of the “live hinge” joints, which in mDR are assumed to be dimensionless pins.

Qualitatively, one observes that the activated states attain structural rigidity with increase in curvature, this can be seen in the SI. Movies. With the dome, by adjusting the stroke length of the unit actuators, its radius can be tailored. If covered with a stretchable fabric, an enclosure is created. With the rhombus-like enclosure, adjustment in stroke length determines the degree of upward rotation, therefore how many unit modules are needed to enclose the space and consequently the interior volume.

The triangular modules in the previous example and square unit modules in this section can be printed individually and connected together post-fabrication in the varying configurations shown. In the activated state, the structure can be treated as a static one, in contrast with the assumed large deformation of tensegrity-like concepts^29^. They can be activated and deactivated without loss in quality, thereby making the designs reversible. Measured geometries and simulation show adequate similarity, allowing for predictable deployment.

## Conclusion

The design and verification of 3D printed, monolithic deployable structures is presented. Activation is achieved using hierarchical design principles where a bistable, monolithically printed Von Mises Truss based unit actuator acts as the basic building block. The stroke length for the unit actuator is maximised. Each design has a flat initial state and is printed with a multi-material 3D printer. By varying the joint material and length of the bistable actuator, numerical simulation and experimentation show that the required activation force can be tuned.

Two groups of hierarchical deployable structures are presented as proof-of-concept designs. The first is a deployable space frame structure where each tetrahedral module is activated from a flat triangle. The second shows the tiling of different square unit modules in a grid to achieve complex, 3D activated structures of varying Gaussian curvature. The deployed states are found with a modified Dynamic Relaxation method. All demonstrated structures have defined load bearing capacity and predictable activated geometry, and are reversible and re-configurable. Future work includes developing computational design methods to explore the design space and optimise active structures.

## Additional Information

**How to cite this article**: Chen, T. *et al*. Integrated Design and Simulation of Tunable, Multi-State Structures Fabricated Monolithically with Multi-Material 3D Printing. *Sci. Rep.* **7**, 45671; doi: 10.1038/srep45671 (2017).

**Publisher's note:** Springer Nature remains neutral with regard to jurisdictional claims in published maps and institutional affiliations.

## Supplementary Material

Supplementary Information

Supplementary Video 1

Supplementary Video 2

Supplementary Video 3

## Figures and Tables

**Table 1:** Geometric comparison between physical specimens (PS) and modified Dynamic Relaxation (mDR) results in mm.

	Unit Module (Fig. 6D,I)						Grid Conf. (Fig. 7A,G)					
	Retracted			Extended			7A			7G		
	PS	mDR	%	PS	mDR	%	PS	mDR	%	PS	mDR	%
*l*_1_	80.5	80.1	0.50	50.0	49.0	2.02	88.5	90.1	1.79	55.0	54.4	1.10
*l*_2_	64.0	64.7	1.09	64.0	63.5	0.78	—	—	—	—	—	—
*h*	16.5	16.7	1.20	30.5	29.9	1.99	48.0	49.8	3.68	62.0	63.9	3.02

With the mDR simulation, the equilibrium states of the grid-based hierarchical designs are found when the total energy drops below *ξ* = 10^−13^ (Fig. 7D–F and J–L). This is achieved for each structure in under 1000 iterations. As shown in Table 1, mDR predicts the nodal coordinates within ±5 of both the square modules and the tiled grid structures.
