# Supplementary material for: Integrated Design and Simulation of Tunable, Multi-State Structures Fabricated Monolithically with Multi-Material 3D Printing
Source: Sci Rep. 2017 Mar 31;7:45671. doi: 10.1038/srep45671 (PMC5374496; doi:10.1038/srep45671)
Supplement: Supplementary Information [file srep45671-s1.pdf]

Integrated Design and Simulation of Tunable, Multi-State Structures Fabricated Monolithically with Multi-Material 3D Printing

Tian Chen[1], Jochen Mueller[1], and Kristina Shea[1,*]

[1]Engineering Design and Computing Laboratory, D-MAVT, ETH Zurich, Switzerland
[*]kshea@ethz.ch

Fabrication Method and Material Characterisation

This section details the fabrication process of the Stratasys Objet500 Connex3 multi-material 3D printer and the mechanical characterisation of the printed materials.

Fabrication Process

The Stratasys Objet500 Connex3 printer achieves multi-material printing by depositing and mixing liquid photopolymers from an array of nozzles in a layer by layer additive fabrication process. UV light cures the recently printed layer before the print platform lowers and the next layer is printed. This allows different materials to be assigned to any region of a design. The printer accepts up to three base materials and one support material. Digital materials (DMs) denote the intermediate materials created at the time of printing by jetting and curing two different base materials at the same location. As uncured materials are in a liquid state, support material is needed under all overhanging geometry.

In this work the two base materials used are TangoBlackPlus(TB+) and VeroWhitePlus(VW+), an elastomer and a rigid plastic respectively. The 12 DMs can be printed by the mixing of these two base materials are also used in the parametric study of the unit actuator. The resulting DMs have varying mechanical properties (See Manuscript Figure 2 for material labels). The characterisation of these properties are described in detail below.

Material Characterisation

Since the simulation of the unit actuator involves large deformation of the complaint joints, a complete stress-strain model for each material is constructed. The material manufacturer provides the Young's Modulus for the seven DMs on the rigid end of the spectrum but not for the flexible ones. Stanković et al.[1] presented the Young's Modulus of all 14 materials, showing large deviations from the manufacturer's specification. The deviations were attributed partly to unknown test conditions.[2] As bistability entails large deflection[3] and finite strain behaviour of the elastomers, quasi-static tensile tests are conducted under constant environmental conditions in this study to obtain complete stress-strain models for each DM.

The following describes the tensile testing procedure that is performed for each material in Manuscript Figure 2. Type V geometry of the ASTM D638-10 test standard for tensile testing of plastics is adopted for all specimens. Three identical test specimens are printed for each material under an ambient temperature of 21 °C. To eliminate the impact of uncontrolled factors on the mechanical properties,[2] each is printed on a separate tray. Aside from the material combination, all other factors are held constant. Tensile tests are conducted on an Instron ElectroPuls 3000 with either a 100 N or a 5000 N load cell, depending on the material tested. Under displacement control, a testing speed of $20\,\mathrm{mm\,min^{-1}}$ is set and the specimens are elongated until fracture.

From the exhibited behaviour, the two dominating materials of the mixing process, TB+ and VW+ can be distinguished. Qualitatively, materials printed with TB+ as the base have a large strain, whereas specimens printed with VW+ as the base exhibits necking and fail subsequently from brittle fracture. From material TB+ to VW+, the strength gradually increases, whereas the fracture strain decreases. In the first group (TB+ to F9895), the fracture strain ranges from 30 % to 174 % and ultimate strength ranges from 0.86 MPa to 10.6 MPa. In the second group (R8530 to VW+), the fracture strain ranges from 8 % to 14 % and ultimate strength ranges from 54 MPa to 69 MPa. In general, the observed properties of the DMs can be grouped around the properties of the two base materials, creating a gap in printable material properties in between.

This bi-modal distribution of the material properties validates the assumption that the mixed materials' properties depend heavily on the base material used. Attention is focused on the TB+ dominating group where the allowable strains are deemed sufficient to accommodate the large deformation induced by the snapping behaviour, and the range of allowable stresses are

sufficient to deliver the desired activation forces. To simplify the material behaviour, each of the 7 TB+ based materials is assumed to be able to be adequately modelled by a multi-linear stress-strain relationship. The elastic Young's Modulus of each material can be calculated by dividing the first stress by the first strain of that material. The control points of each of the material are listed in Table 1.

Table 1. Constitutive model of materials TB+ to F9895 and VW+ for physically non-linear analysis

Name	E_{nom} MPa	σ_1 MPa	ε_1 -	σ_2 MPa	ε_2 -	σ_3 MPa	ε_3 -
TB+	0.486	0.850	1.75	-	-	-	-
F9840	1.36	1.50	1.10	1.50	2.00	-	-
F9850	2.65	2.65	1.00	2.65	2.00	-	-
F9860	22.5	0.45	0.02	0.75	0.05	4.0	0.85
F9870	52.5	1.00	0.02	1.60	0.05	4.8	0.60
F9885	125	2.50	0.02	3.75	0.05	7.0	0.45
F9895	375	7.50	0.02	9.50	0.05	10.5	0.26
VW+	2500	50.0	0.02	70.0	0.04	65.0	0.05

Table 1 shows that TB+ can be described by a linear relationship. F9840 and F9850 can be described bi-linearly; the strain value at the first control point of these materials, 200 %, are much higher than that of the stiffer materials. F9860 to F9895 are described tri-linearly, the second strain point is set at 5 % to describe the softening effect and the third strain point is set at rupture. The behaviour of VW+ is distinct from the rest as it belongs to the rigid material group and follows the behaviour shown in Manuscript Figure 2C. These values are incorporated in the non-linear Finite Element Analysis (FEA) of the actuators.

Unit Actuator: Design, Simulation & Experimentation

This section details the geometric parameters needed for modelling the bistable unit actuator as shown in Manuscript Figure 1C. A parametric study of the proposed unit actuator incorporating both simulation and experimentation is described.

Geometry of the Unit Actuator

The geometry of the design shown in Manuscript Figure 1C is listed in Table 2. It is important to note that the trusses in the actuator are dimensioned to be the minimum feature size that the Connex3 printer can reliably print. The remaining dimensions are sized accordingly.

Table 2. Dimensions defining the actuator design shown in Manuscript Figure 1C

Parameter	Value	Parameter	Value
a	6.825 mm	w_0	4.50 mm
b	6.250 mm	L	4.50 mm
c	6.675 mm	l_1	0.25 mm
d	5.00 mm	l	1.0, 0.75, 0.50 mm
e	1.25 mm	t	0.50 mm
f	5.00 mm	s	0.50 mm
g	2.00 mm	α	45°
h	2.00 mm		

Parametric study of the Unit Actuator

The parametric study of the unit actuator is conducted both through simulation and experimentation. It focuses on the actuator's overall behaviour and activation force. The initial configuration of the actuator at fabrication, i.e. retracted or extended, is included as an additional parameter. Along with three different joint lengths ($l = 1.0, 0.75, 0.50$ mm) and seven different joint materials in the TB+ group, the parametric variations lead to a total of 42 models shown in Figure 1. The specimen number used in Manuscript Figure 4D can be referenced in Table 3.

A physically and geometrically non-linear implicit simulation using beam elements (B32 in Abaqus) is performed to predict the behaviour of a unit actuator. The beam elements are placed at the centre line of the real geometry as shown in Figure 2A.

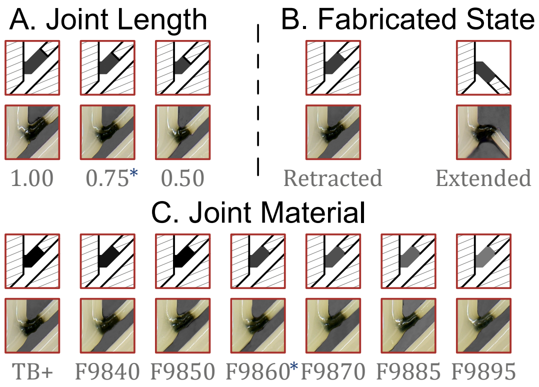

Figure 1. Joint length, initial state, and joint material are the design parameters studied. These are shown by focusing on one actuator joint. * denotes the benchmark model.

Table 3. Specimen number, and the corresponding joint dimension and material. * denotes the specimens whose results are plotted in Manuscript Figures 4ABC, † denotes the benchmark model

	1.0 mm	0.75 mm	0.50 mm
TB+	1	2*	3
F9840	4	5*	6
F9850	7	8*	9
F9860	10*	11*†	12*
F9870	13	14*	15
F9885	16	17*	18
F9895	19	20*	21

Where there is a physical separation, a link element is added to better reassemble reality. A vertical displacement of ±10 mm is applied to Point A; Point C is pin jointed. Symmetry conditions are imposed on Points B. The plastic materials model detailed in Table 1 are incorporated into the analysis.

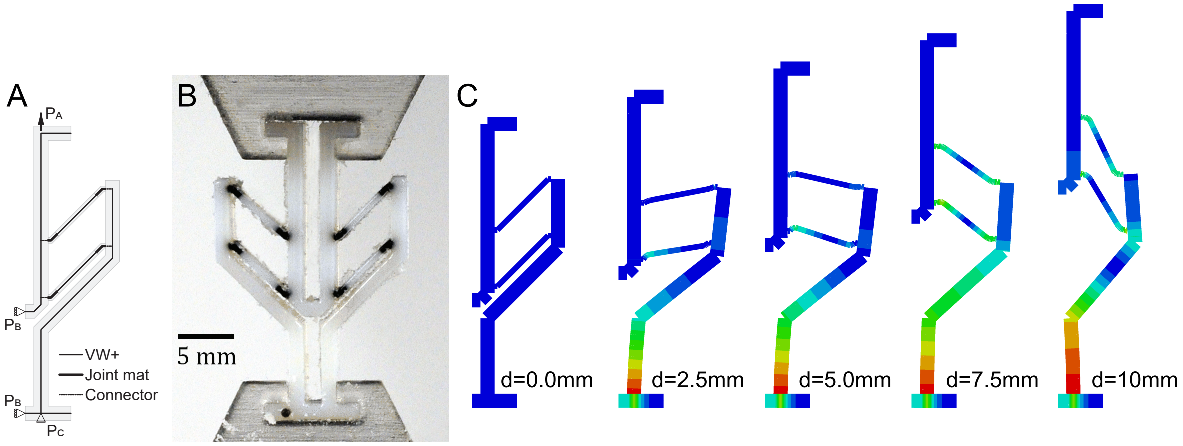

Figure 2. A. shows the simulation model with important points labelled. B shows the load testing setup including the printed grippers to match the simulation boundary conditions. C shows the deformation behavior as displacement increases, Von Mises stress contour is shown as a color overlay.

To validate the simulation results, five set of all 42 physical specimens are fabricated and load tested under tension or compression (Figure 2B) depending on the initial states. An Instron ElectroPuls 3000 dynamic testing machine is used to apply a controlled displacement. To ensure that the boundary conditions remain invariant across all models and are identical to the numerical simulations, a set of two customised grippers are printed to hold the specimens. A prescribed displacement of

$\pm 10\,\mathrm{mm\,min^{-1}}$ is set depending on the initial state. Both the actual displacement and the reaction force are recorded for each specimen at a sampling rate of $10\,\mathrm{Hz}$. The results are presented and discussed in the paper.

Simulation using Modified Dynamic Relaxation

Four modifications on the classic formulation of Dynamic Relaxation are introduced in this work. One, no external force is applied. Instead, the rest length of the springs which contains unit actuator(s) has the stroke length added or subtracted from it. This is a form of pre-stressing that makes the 2D configurations unstable. Two, the force density matrix is updated at every iteration as a result of length change. Three, this unstable equilibrium must be perturbed to assume the 3D shapes. In physical systems, the perturbation usually takes the form of geometric imperfections. In the simulation, an upward initial velocity is added to an arbitrary node to trigger the snapping behaviour. Four, no nodal fixity is assumed, i.e. all nodal positions are updated at every iteration. Rigid body motion may occur as a consequence. Should static boundary conditions be necessary, the following derivation must be changed to account for only the free DOFs.

The algorithm summarised below is grouped into the initialisation phase and the time iteration phase. To initialise the routine, step one is to construct the connectivity matrix of the structure consisting of n vertices, and m_e edges.

$$C_{k,p} = \begin{cases} \mathrm{sign}(j-p) & \text{if } p=i; \\ \mathrm{sign}(i-p) & \text{if } p=j; \quad (p=1,2,\ldots,n) \\ 0 & \text{otherwise.} \end{cases} \tag{1}$$

where i and j are the nodes connecting the kth member. k and p iterates through every element and every node respectively. To obtain the activated length of each member, a stroke length array is defined as a modifier of the initial length. Note that to obtain all unique activated states, one must iterate through all combinations of the bistable actuators within a structure. The upper bound of all unique states is $S = 2^n$ where n is the number of bistable actuators. Redundancy results when two or more actuators are connected in series, the overall stroke length is independent of which actuator in the series is activated. The length modifier, $l_{\mathrm{stroke},k}$, of the kth member is calculated as:

$$l_{\mathrm{stroke},k} = n_{\mathrm{s},k} n_{\mathrm{a},k} s_k \quad (k=1,2,\ldots,m_\mathrm{e}) \tag{2}$$

where $\mathbf{n}_\mathrm{a}$ contains the number of actuators in each edge and $\mathbf{s}$ is the stroke length of each actuator. The initial state of the actuators is defined as follows,

$$n_{\mathrm{s},k} = \begin{cases} +1 & \text{retracted;} \\ -1 & \text{extended;} \\ 0 & \text{otherwise.} \end{cases} \tag{3}$$

Let $\mathbf{x}^{t=0}$ be the coordinates of the initial nodes, we can calculate the activated length of the structure as follows. The array is rearranged into a diagonal matrix for later operations.

$$\mathbf{L}_\mathrm{a} = \mathrm{diag}\left\{\|\mathbf{C}\mathbf{x}^{t=0}\| + \mathbf{l}_\mathrm{stroke}\right\} \tag{4}$$

The iteration phase starts by setting $t = 0$. The member lengths are then calculated and collected into a diagonal matrix.

$$\mathbf{L}^t = \mathrm{diag}\|\mathbf{C}\mathbf{x}^t\| \tag{5}$$

The definition of the force density matrix Q is adapted from.[4] $\mathbf{E}$ and $\mathbf{A}$ are diagonal matrices containing the stiffness and the cross-sectional area of the members respectively. These can be identity matrices if all members are identical in mechanical properties.

$$\mathbf{Q}^t = \mathbf{E}\mathbf{A}(\mathbf{L}_\mathrm{a} - \mathbf{L}^t)^{-1} \tag{6}$$

Next the residual force $\mathbf{r}$ is calculated at every node (see Schek[5] for derivation). Note that external force is zero and omitted from the equation.

$$\mathbf{r}^t = -\mathbf{C}^\mathrm{T}\mathbf{Q}^t\mathbf{C}\mathbf{x}^t \tag{7}$$

Velocity is updated by calculating nodal acceleration, and incorporating viscous and kinetic damping (see Adriaenssens et al.[6] for derivation) with $\mathbf{A^D}$ and $\mathbf{B^D}$. Initial velocity perturbation is incorporated in $\mathbf{v}^{t=0}$. Mirrored activated states can be found by changing this perturbation.

$$\mathbf{v}^{t+\Delta t} = \mathbf{A^D}\mathbf{v}^t + \mathbf{B^D}\Delta t\frac{\mathbf{r}^t}{\mathbf{m}} \tag{8}$$

Where **m** are the fictitious nodal mass. The node coordinates are updated as follows,

$$\mathbf{x}^{t+\Delta t} = \mathbf{x}^{t} + \mathbf{v}^{t+\Delta t}\Delta t \tag{9}$$

Iteration stops when the total energy of the system approaches a small number ξ, as this implies that there is no velocity in the system and that none of the springs are stressed.

$$\sum_{k=1}^{m_\mathrm{e}} \left(\frac{m_k}{2} {v_k}^2 + \frac{E_{k,k} A_{k,k}}{2 L_{k,k}} \left(L_{0k,k} - L_{k,k} \right)^2 \right) \leq \xi \tag{10}$$

Supplementary Image

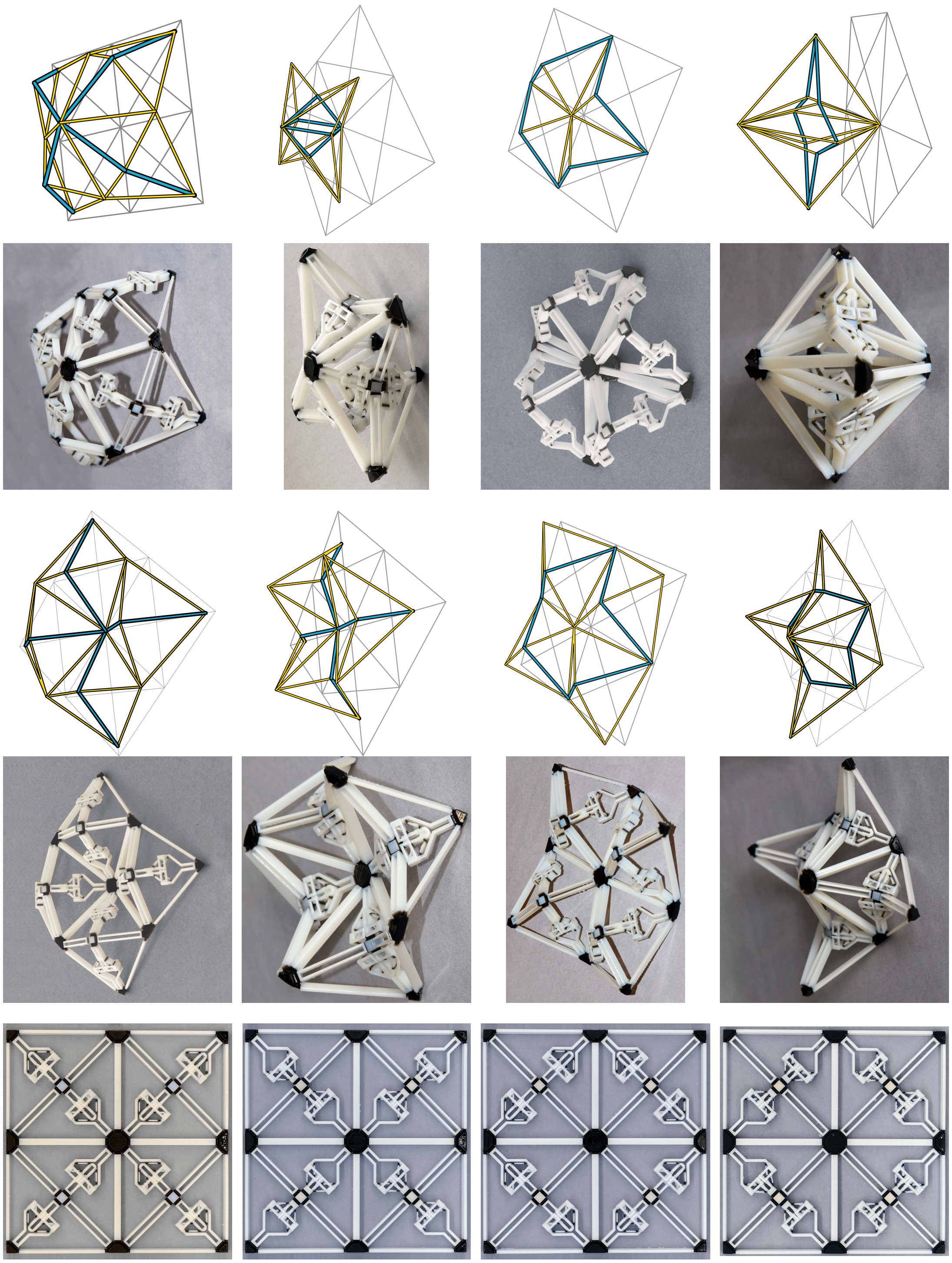

Activated geometries of all grid-based designs

Supplementary Videos

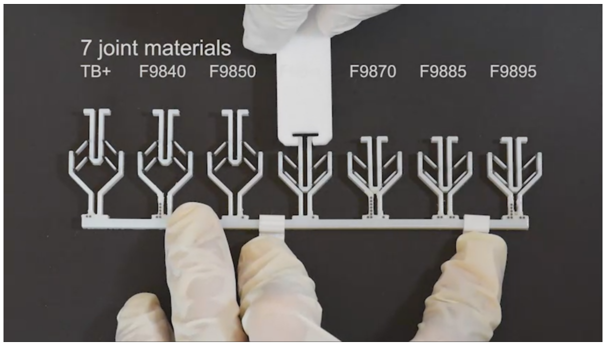

Video 1 - Actuation of the bistable unit actuator: This video shows the activation of unit actuators with different joint material, joint length and initial configuration.

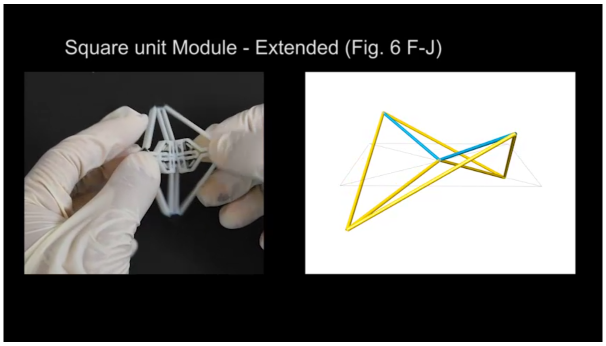

Video 2 - Actuation and simulation of the square unit module: This videos shows physical activation of the square unit modules and its simulation. By switching the initial configurations, we achieve positive or negative Gaussian curvatures.

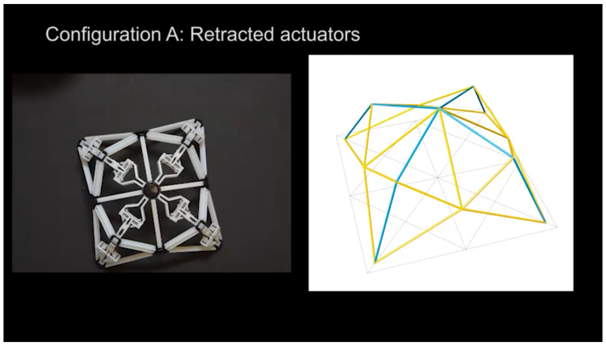

Video 3 - Actuation and simulation of tiled surfaces: This videos shows physical activation of the tiled surfaces and the simulation counterpart. In particular, load bearing and multi-state characteristics are demonstrated.

References

1. Stankovic, T., Mueller, J., Egan, P. & Shea, K. A Generalized Optimality Criteria Method for Optimization of Additively Manufactured Multimaterial Lattice Structures. *Journal of Mechanical Design* **137**, 111405 (2015). DOI 10.1115/1.4030995.
2. Mueller, J., Shea, K. & Daraio, C. Mechanical properties of parts fabricated with inkjet 3D printing through efficient experimental design. *Materials & Design* **86**, 902–912 (2015). URL http://dx.doi.org/10.1016/j.matdes.2015.07.129. DOI 10.1016/j.matdes.2015.07.129.
3. Howell, L. L., Rao, S. S. & Midha, A. Reliability-Based Optimal Design of a Bistable Compliant Mechanism. *Journal of Mechanical Design* **116**, 1115–1121 (1994). URL http://link.aip.org/link/?JMD/116/1115/1.
4. Zhang, J. Y. & Ohsaki, M. Adaptive force density method for form-finding problem of tensegrity structures. *International Journal of Solids and Structures* **43**, 5658–5673 (2006). DOI 10.1016/j.ijsolstr.2005.10.011.
5. Schek, H. J. The force density method for form finding and computation of general networks. *Computer Methods in Applied Mechanics and Engineering* **3**, 115–134 (1974). DOI 10.1016/0045-7825(74)90045-0.
6. Adriaenssens, S., Barnes, M., Harris, R. & Williams, C. Dynamic relaxation. In Adriaenssens, S., Block, P., Veenendaal, D. & Williams, C. (eds.) *Shell Structures for Architecture: Form Finding and Optimization*, chap. 8, 89–101 (Routledge, Abingdon, Oxon, 2014), 1st edn. URL http://www.tandfebooks.com/isbn/9781315849270.
